# Predictive performance of regression models to estimate Chlorophyll-a concentration based on Landsat imagery

**DOI:** 10.1371/journal.pone.0205682

**Published:** 2018-10-12

**Authors:** Miguel Ángel Matus-Hernández, Norma Yolanda Hernández-Saavedra, Raúl Octavio Martínez-Rincón

**Affiliations:** 1 Centro de Investigaciones Biológicas del Noroeste, La Paz, Baja California Sur, México; 2 CONACYT—Centro de Investigaciones Biológicas del Noroeste, La Paz, Baja California Sur, México; Beijing Normal University, CHINA

## Abstract

Chlorophyll-a (Chl-a) concentration is a key parameter to describe water quality in marine and freshwater environments. Nowadays, several products with Chl-a have derived from satellite imagery, but they are not available or reliable sometimes for coastal and/or small water bodies. Thus, in the last decade several methods have been described to estimate Chl-a with high-resolution (30 m) satellite imagery, such as Landsat, but a standardized method to estimate Chl-a from Landsat imagery has not been accepted yet. Therefore, this study evaluated the predictive performance of regression models (Simple Linear Regression [SLR], Multiple Linear Regression [MLR] and Generalized Additive Models [GAMs]) to estimate Chl-a based on Landsat imagery, using *in situ* Chl-a data collected (synchronized with the overpass of Landsat 8 satellite) and spectral reflectance in the visible light portion (bands 1–4) and near infrared (band 5). These bands were selected because of Chl-a absorbance/reflectance properties in these wavelengths. According to goodness of fit, GAM outperformed SLR and MLR. However, the model validation showed that MLR performed better in predicting log-transformed Chl-a. Thus, MLR, constructed by using four spectral bands (1, 2, 3, and 5), was considered the best method to predict Chl-a. The coefficients of this model suggested that log-transformed Chl-a concentration had a positive linear relationship with bands 1 (coastal/aerosol), 3 (green), and 5 (NIR). On the other hand, band 2 (blue) suggested a negative relationship, which implied high coherence with Chl-a absorbance/reflectance properties measured in the laboratory, indicating that Landsat 8 images could be applied effectively to estimate Chl-a concentrations in coastal environments.

## Introduction

Coastal environments are highly productive and complex marine ecosystems because they show the interaction of various natural and anthropogenic phenomena that provide an important source of nutrients for phytoplankton and aquatic organisms, as well as for various human activities. Nevertheless, during the last decades, studies have demonstrated that these water bodies have been under significant stress due to anthropogenic alterations and climate variations that are increasingly frequent events, such as algal blooms [1,2]. In these environments, Chl-a has been considered as one of the most important parameters for measuring water quality, so it can be used as an indicator of ecosystem health [3,4].

The concentration of Chl-a varies spatially in coastal regions, and conventional methods for point-to-point studies are expensive, require time, and are usually spatially incomplete [5,6]. Therefore, several techniques have been proposed recently to use remote sensors as a viable option for monitoring environmental parameters at local spatial scales through images with high spatial resolution. Several algorithms have been developed to measure Chl-a based on the relationship that exists between the reflectance of different wavelengths from sensors specifically designed for monitoring Chl-a in marine environments, such as Coastal Zone Color Scanner (CZCS) with a spatial resolution of 825 m [7]; Sea-Viewing Wide Field-of-View Sensor (SeaWiFS) of 1130 m [8,9]; Medium Resolution Imaging Spectrometer (MERIS) of 300 m [10]; and Moderate Resolution Imaging Spectroradiometer (MODIS) of 250 m, 500 m and 1000 m [11,12]. Nonetheless, several difficulties have been reported when performing adequate monitoring of these environments, among which those of low resolution can only be applied effectively in homogeneous open sea areas but not for spatially complex coastal environments, such as bays or estuaries that require a higher spatial resolution for their study [1,5].

Landsat satellite series have provided a temporary record of multispectral images of the longest land surface in history since 1972, registry widely used for several governmental, public, and private applications [13]. The last satellite of this series is Landsat 8, which consists of two sensors, one called Operational Land Imager (OLI) and the other one Thermal Infrared Sensor (TIRS). Both of them obtain data jointly to provide land surface images, including coastal regions, polar ice, islands, and continental zones [14]. Although this satellite was designed for the study of terrestrial processes and limited to spectral and temporal resolution for oceanic applications, its high spatial resolution (30 m) makes it ideal for applications in small water bodies [15].

Recent studies have demonstrated the broad potential of Landsat images in lakes and coastal environments (bays and inlets) based on the existent correlations between band reflectance and different water properties, such as: Secchi disk transparency (SDT)[16–18]; concentration of suspended sediments [2,19–21]; turbidity [18,22–24]; studies of colored dissolved organic matter (CDOM) [15,23,25,26] and macroalgal blooms [27]; and quantifying Chl-a concentrations [18,28–32]. Therefore, the main objective of this study was to select the best model to estimate Chl-a concentration from *in situ* measurements and Landsat 8 satellite images in the Bahía de La Paz, Mexico by using multiple linear regression models to evaluate their possible application in monitoring Chl-a concentration in coastal water bodies.

## Materials and methods

### Study area

**Fig 1:**
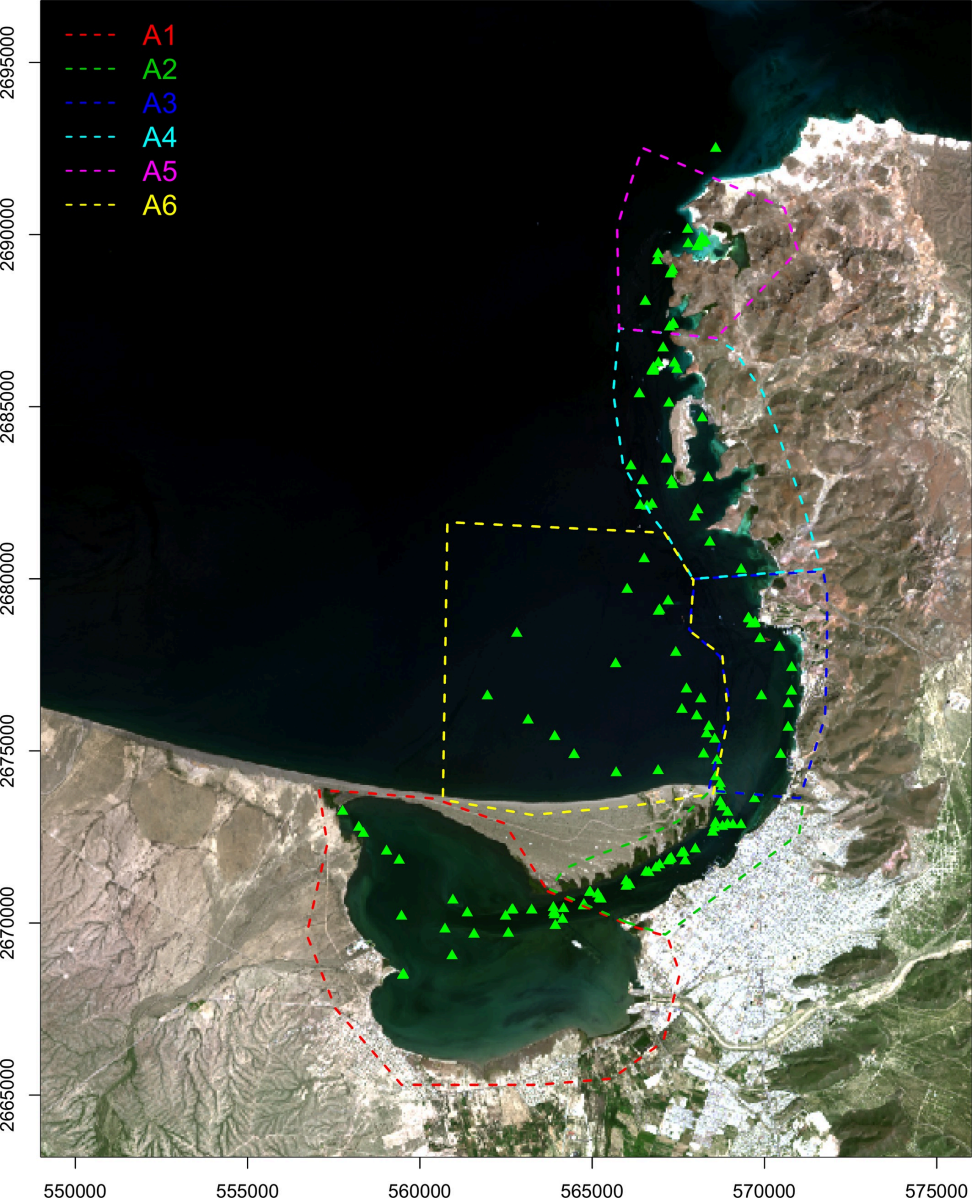
Map of the geographical location of the study area. Distribution of the sampling sites (green triangles) and arbitrary areas (polygons) used for time series analysis. Map generated in programming language R using Landsat 8 image from 2016-09-09.

The study area is located within the Bahía de La Paz on the western coast of Baja California Sur, Mexico between 24° 09' and 24° 47' N, and 110° 45' and 110° 18' W (Fig 1). It is a coastal water body of about 90 km long, 60 km wide and 4500 km^2^ with two water mouths that connect it with the western region of the Gulf of California. The main water mouth is wide and 300 m in depth located to the northwest while to the east, the water mouth (small mouth or the San Lorenzo Canal) is narrow and shallow associated with 20-m deep channels [33,34].

The Ensenada de La Paz is a coastal lagoon located in the southern part of the Bahía de La Paz between 24° 06' and 24° 11' N, and 110° 19' and 110° 25' W. It is a protected coastal water body separated from the Bahía de La Paz by a marine sandy barrier called "El Mogote", approximately 11 km long in east-western direction and 2.7 km in its widest part [35]. Ensenada de La Paz is 12 km in length, 5 km in width in an area of 45 km^2^ with respect to sea level average. Morphologically speaking, the water mouth is formed by two parallel channels in their connection with the Bahía de La Paz of approximately 4 km in length and 0.6 km in width in total with an average depth of 7.0 m [36].

### Field data collection

The *in situ* data collection was done synchronously with the overpass of Landsat 8 satellite, which passes by this zone every 16 days at approximately 17:47 UTC. Thus, field trips were made two hours before and after 17:47 UTC to avoid the effect of Chl-a variability related to tides and local currents. The Chl-a concentration was measured near the surface (~ 50 cm deep), taken with the multi-parameter sensor RBRmaestro model XRX-420 produced by RBR Ltd in Ottawa, Canada. No specific permissions were required for our study locations/activities since Chl-a data was collected in non-protected or private locations of the study area.

Twelve field campaigns were performed for over one year of monitoring due to bad weather (mainly high cloudiness), and field trips did not take place some dates of the period of study. Table 1 shows the dates of the field trips made, as well as some descriptive statistics of Chl-a measured *in situ* for the six arbitrary areas (polygons) used for time series analysis (see Fig 1 for details).

**Table 1:** Descriptive statistics of *in situ*-measured Chlorophyll-a concentrations (μg*l^-1^).

Date	A1			A2			A3		
	Min	Mean	Max	Min	Mean	Max	Min	Mean	Max
2016-08-24				0.33	0.44	0.55	0.18	0.19	0.20
2016-09-09	1.37	1.37	1.37	0.43	0.72	1.34	0.37	0.51	0.61
2016-09-25	0.95	1.14	1.41	0.61	0.79	1.05			
2016-10-27	0.41	0.58	0.79	0.30	0.33	0.38			
2016-11-28	0.58	0.58	0.58	0.51	0.54	0.56			
2017-01-31	0.42	0.47	0.52	0.39	0.39	0.39	0.52	0.52	0.52
2017-02-16	0.37	0.37	0.37	0.38	0.38	0.38	0.36	0.36	0.36
2017-03-20	0.51	0.85	1.04	0.59	0.75	0.99	0.33	0.33	0.34
2017-04-05	0.33	0.33	0.33	0.39	0.44	0.47	0.19	0.19	0.19
2017-04-21	0.50	0.59	0.68	0.52	0.52	0.53			
2017-05-23	0.35	0.47	0.59	0.26	0.31	0.37	0.33	0.33	0.33
2017-06-08	2.12	2.12	2.12	2.11	2.23	2.37	1.42	2.10	2.71
Date	A4			A5			A6		
	Min	Mean	Max	Min	Mean	Max	Min	Mean	Max
2016-08-24	0.16	0.28	0.66				0.25	0.25	0.25
2016-09-09	0.61	0.61	0.61						
2016-09-25							0.17	0.24	0.41
2016-10-27	0.18	0.18	0.18				0.20	0.23	0.27
2016-11-28	0.40	0.41	0.41	0.38	0.42	0.45	0.34	0.36	0.37
2017-01-31	0.42	0.42	0.42	0.33	0.36	0.41	0.43	0.43	0.43
2017-02-16	0.49	0.60	0.71	0.64	0.66	0.68	0.25	0.25	0.25
2017-03-20	0.49	0.51	0.53	0.43	0.50	0.58			
2017-04-05	0.24	0.28	0.33	0.36	0.36	0.36	0.22	0.22	0.22
2017-04-21	0.16	0.16	0.17	0.15	0.17	0.20	0.18	0.25	0.40
2017-05-23	0.14	0.17	0.24	0.14	0.17	0.23	0.22	0.22	0.22
2017-06-08	0.46	0.94	1.52						

Min, Minimum; Max, maximum; SD. A1 to A6 arbitrary areas (see Fig 1 for details).

### Satellite data (Landsat 8 images)

Landsat 8 Level 1 data products were used in this study, which were included in the Landsat 8 OLI/TIRS C1 Level-1 data set and downloaded from the US Geological Survey server (USGS, https://www.usgs.gov) using Earth Explorer platform (https://earthexplorer.usgs.gov). Landsat 8 has two sensors onboard Operational Land Imager (OLI) and Thermal Infrared Sensor (TIRS). In total these sensors had 11 spectral bands, nine of the OLI sensor and two of the TIRS sensor. The spatial resolution of bands 1–7 and 9 was 30 m; band 8 (panchromatic) was 15 m and 100 m for bands 10–11. In this study, the following spectral bands of the visible light portion and near infrared (NIR) were used; B1 (coastal/aerosol: 0.435–0.451 μm); B2 (blue: 0.452–0.512 μm); B3 (green: 0.533–0.590 μm); B4 (red: 0.636–0.673 μm); and B5 (NIR: 0.851–0.879 μm).

The study area was in the Landsat ID scene: LC8034043 (Path = 34, Row = 43). The images were acquired from 2016-08-24 to 2017-06-08, and only were those without cloud cover selected and cropped to highlight the study area (Fig 1). Landsat 8 images were imported and processed with the *raster* library [37] from programming language R [38] version 3.3.2 to obtain water pixel remote sensing reflectance of each one of the selected bands, which was calculated by using the equations in Landsat 8 user manual [13].

### Statistical modeling

This study used linear regression (LR) and generalized additive models (GAM) to develop a model to estimate Chl-a concentrations from *in situ* data and spectral reflectance of Landsat 8 bands 1–5 images. LR explored the linear relationship between response and predictor variables; GAM explored linear or non-linear relationships between response and predictor variables throughout smooth functions (e.g. thin plate regression spline). Assuming that error terms (residuals) were independent of the predictor variables, normally distributed with mean 0 and homoscedastic [39, 40].

For modeling we used 147 records of log-transformed Chl-a as response variable and spectral bands of the visible light portion (B1 [coastal/aerosol], B2 [blue], B3 [green], and B4 [red]) and near infrared (NIR, B5) as predictor variables. Values of Chl-a concentration were logarithmically transformed and used as dependent variable because some authors have widely described that chlorophyll showed a non-linear relationship with Landsat bands [41,42].

LR models were constructed using a single predictor variable or more than one to highlight the number of predictor variables; we named Simple Linear Regression (SLR) when one predictor variable was used in the model and Multiple Linear Regression (MLR) when two or more predictor variables were used. All data processing and statistical models were conducted in R [38]; *mgcv* library was used for GAM [43].

A different band combination was tested for SLR, MLR, and GAM since previous studies on Chl-a estimation from Landsat imagery suggested that addition, multiplication, proportion, or quadratic transformation of bands 1–5 gave good results in SLR [5,23,42,44,45]. Up to two bands were combined through permutation to be used as predictor variables in SLR. Thus, for SLR we tested a total of 250 different models (S1 Table). For MLR we used band combinations with two, three, four and five predictor variables using spectral bands 1–5 without transformation. For MLR we tested a total of 26 different models (S2 Table). For GAM we used each single band and band combinations using two, three, four and five predictor variables and tested a total of 31 different models (S3 Table). To date, GAM had not been used to estimate Chl-a from Landsat imagery.

In general, the models can be represented as follows:

$$y_{i}=\alpha +\beta X+\varepsilon_{i}$$ (1)

$$y_{i}=\alpha +\beta_{1}X_{1}+\beta_{2}X_{2}+\beta_{3}X_{3}+\cdots +\beta_{p}X_{p}+\varepsilon_{i}$$ (2)

$$y_{i}=\alpha +f_{1}X_{1}+f_{2}X_{2}+f_{3}X_{3}+\cdots +f_{p}X_{p}+\varepsilon_{i}$$ (3)

where *y*_*i*_ was the expected value of log-transformed Chl-a concentration (μg*l^-1^); α = intercept; ß_p_ were the coefficients of predictor variables (*X*_*p*_), which were bands 1–5; and *f*_*p*_ were smooth functions (thin plate regression spline) of the covariates; ε_i_ the error terms (residuals) were independent of X and they were assumed to be normally distributed with mean 0 and homoscedasticity. GAMs were used with Gaussian error distribution and identity link function.

### Goodness of fit and predictive performance

The quality of model fit was assessed using the R squared (R^2^) and adjusted R squared (adj. R^2^). These statistics were used to describe the proportion of variance explained and could take values between 0 and 1. The difference between R^2^ and adj. R^2^ relied in that the latter used a penalization based on the number of parameters (predictor variables); this variation of R^2^ was used because it had been demonstrated that R^2^ always increased when a new predictor variable was added to the model, causing an overparameterization of models. Therefore, adj. R^2^ was preferred for models with two or more predictor variables.

Data splitting is an effective method for evaluating predictive performance of a given model in which a portion of the data is used to estimate model coefficients, and the remainder of the data is used to measure prediction accuracy of the model. In this study data were arbitrarily separated into two subsets, training data (2016-08-24, 2016-09-09, 2016-09-25, 2016-10-27, 2016-11-28, 2017-01-31, 2017-02-16 and 2017-03-20) and test data (2017-04-05, 2017-04-21, 2017–05–23 and 2017-06-08). Thus, after fitting models on the training data, their performance was measured against test data.

Predictive performance of the models was evaluated using root-mean-square error (RMSE) and correlation coefficient (R) (S4–S6 Tables); these values were calculated using observed and predicted data from the test dataset. Predicted data was computed using coefficients or smooth functions of all models; thus RMSE of all models was on the same scale (log-transformed Chl-a). RMSE was defined as:

$$RMSE=\sqrt{\frac{1}{n}\sum_{i=1}^{n}(X_{i}^{observed}-X_{i}^{predicted}{)}^{2}}$$

Finally, to evaluate model assumptions, we analyzed residuals of the best fitted SLR, MLR, and GAM, respectively to identify if they were normally distributed, homoscedastic and had the presence of outliers.

### Chlorophyll-a estimation from Landsat 8

Using the best fitted model and all Landsat 8 scenes available and cloud free for the study area, we obtained the estimated log-transformed Chl-a for the period May 2013—October 2017; log-transformed Chl-a was returned to its original scale by applying the exponential function. To describe spatial and temporal variability of estimated Chl-a, we analyzed time-series data on six arbitrary areas (polygons) in the study area (Fig 1). These polygons were defined considering geographical features: (1) a semi-closed coastal lagoon (Ensenada de La Paz); (2) the channel that communicates Ensenada de La Paz with Bahía de La Paz; (3) the northernmost part of the city of La Paz; (4) the port city; (5) the second largest presence of mangroves of the study area; and (6) the southernmost portion of Bahía de La Paz locally known as El Mogote.

## Results

### *in situ* Chlorophyll-a data

Table 1 shows the descriptive statistics of Chl-a concentration measured during field trips, which ranged from 0.136 to 2.714 μg*l^-1^; the highest average value (1.652 μg*l^-1^) was recorded in 2017-06-08 and the lowest one (0.252 μg*l^-1^) in 2017-05-23; the highest values were recorded during a red tide event. As shown in Table 1, average Chl-a values were usually lower than 0.7.

**Fig 2:**
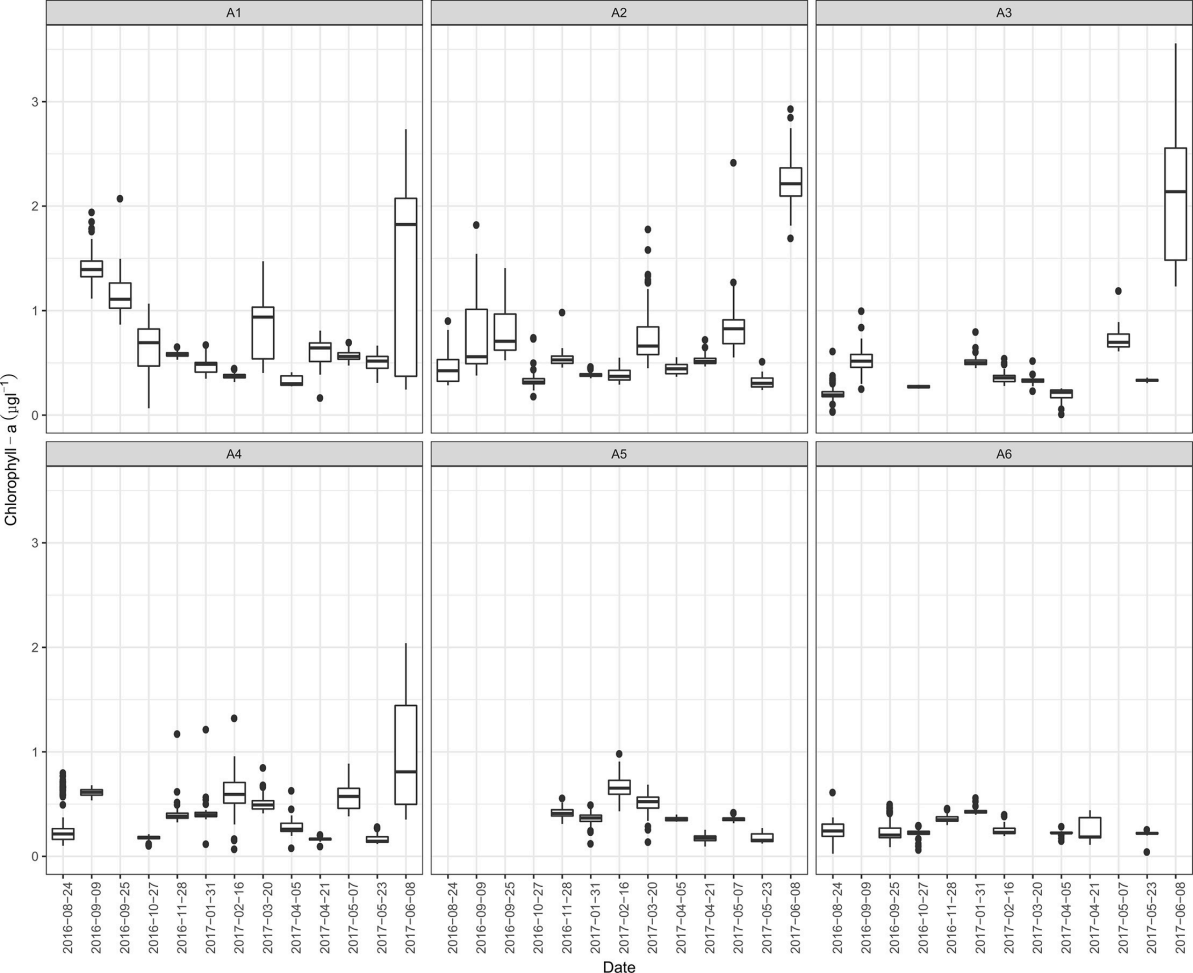
Observed chlorophyll-a values during the survey period. Solid lines represent medians; boxes the interquartile ranges; whiskers minimum and maximum or 1.5 times the interquartile range (when outliers were present); points represent the outliers. A1-A6 arbitrary areas (see Fig 1 for details).

Fig 2 shows spatial and temporal variability of the observed Chl-a during the survey period. As it can be observed, Chl-a values were usually higher in area 1 and 2 with respect to others. The highest ones were recorded in June 8, 2017 in areas 1–3 and corresponded to the red tide event.

### Selection of the best fitted model

A total of 307 models were constructed and evaluated to identify which of them could explain the highest variance proportion of log-transformed Chl-a, inferred from R^2^ and adjusted R^2^. Table 2 shows the three best fitted SLR, MLR, and GAM, respectively. SLR resulted in the lowest proportion of variance explained (R^2^ = 0.000–0.542; adj. R^2^ = -0.009–0.538); MLR in a higher proportion of variance explained (R^2^ = 0.061–0.764; adj. R^2^ = -0.044–0.753) while GAM resulted in the highest proportion of variance explained (adj. R^2^ = 0.216–0.854). The SLR with the highest R^2^ and adjusted R^2^ was the ratio between B4 (red) and B1^2^ (coastal/aersol), explaining 54.2% of total variance. The MLR with the highest R^2^ and adjusted R^2^ was that which included the five spectral bands, explaining 76.4% of total variance. The GAM with the highest adjusted R^2^ was that which included four spectral bands (B1 [coastal/aerosol], B2 [blue], B3 [green], and B4 [red]), explaining 88.7% of total deviance.

**Table 2:** Goodness of fit of the three best fitted SLR, MLR, and GAM, respectively, for log-transformed Chl-a estimation.

Model	R^2^	adj.R^2^
**Simple Linear Regression**		
y = 1.84–6.54*(B1^2^/B4)	0.506	0.502
y = -3.6 + 1.09*(B4/B1^**2**^**)**	**0.542**	**0.538**
y = -3.06 + 5.55*(B4/B2)	0.477	0.473
**Multiple Linear Regression**		
y = 0.94 + 88.45*B1–194.77*B2 + 97.55*B3 + 10.79*B4	0.735	0.725
y = 1.54 + 79.56*B1–191.62*B2 + 102.22*B3 + 13.17*B5	0.757	0.748
y = 1.05 + 103.37*B1–221.63*B2 + 119.1*B3–19.09*B4 + 21.39*B5	**0.764**	**0.753**
**Generalized Additive Models**		
**y = f(B1) + f(B2) + f(B3) + f(B4)**		**0.854**
y = f(B1) + f(B2) + f(B3) + f(B5)		0.848
y = f(B1) + f(B2) + f(B3) + f(B4) + f(B5)		0.847

R^2^, Coefficient of determination; adj. R^2^, adjusted coefficient of determination. In bold the best fitted SLR, MLR, and GAM, respectively.

### Predictive performance

As mentioned in Methods, predictive performance was evaluated using Pearson coefficient of correlation (R) and root-mean-square error (RMSE) obtained from predictions of the training model on an independent data set. As shown in Table 3, the SLR with the highest R and lowest RMSE was the model with the ratio between B4 and B1^2^; the MLR with the highest R and lowest RMSE was the one that included four bands (B1 [coastal/aerosol], B2 [blue], B3 [green], and B5 [NIR]); and the GAM with the highest R and lowest RMSE was the one that included B1, B2, and B3. These results indicated that three modeling approaches could predict log-transformed Chl-a with high accuracy.

**Table 3:** Predictive performance of the three best fitted SLR, MLR, and GAM, respectively, applied for log-transformed Chl-a estimation.

Model	R	MSRE
**Simple Linear Regression**		
y = 1.84–6.54*(B1^2^/B4)	0.791	0.288
y = -3.6 + 1.09*(B4/B1^**2**^**)**	**0.858**	**0.255**
y = -3.06 + 5.55*(B4/B2)	0.849	0.303
**Multiple Linear Regression**		
y = 1.48 + 81.53*B1–187.8*B2 + 98.22*B3	0.856	0.209
y = 2.13 + 66.02*B1–174.51*B2 + 95.85*B3 + 5.23*B5	**0.875**	**0.191**
y = 1.39 + 95.99*B1–211.35*B2 + 117.22*B3–22.85*B4 + 13.2*B5	0.866	0.194
**Generalized Additive Models**		
**y = f(B1) + f(B2) + f(B3)**	**0.835**	**0.239**
y = f(B2) + f(B3) + f(B4)	0.821	0.286
y = f(B1) + f(B2) + f(B3) + f(B4)	0.798	0.274

R, Pearson coefficient of correlation; RMSE, root-mean-square error. In bold SLR, MLR, and GAM, respectively, with the highest predictive performance.

**Fig 3:**
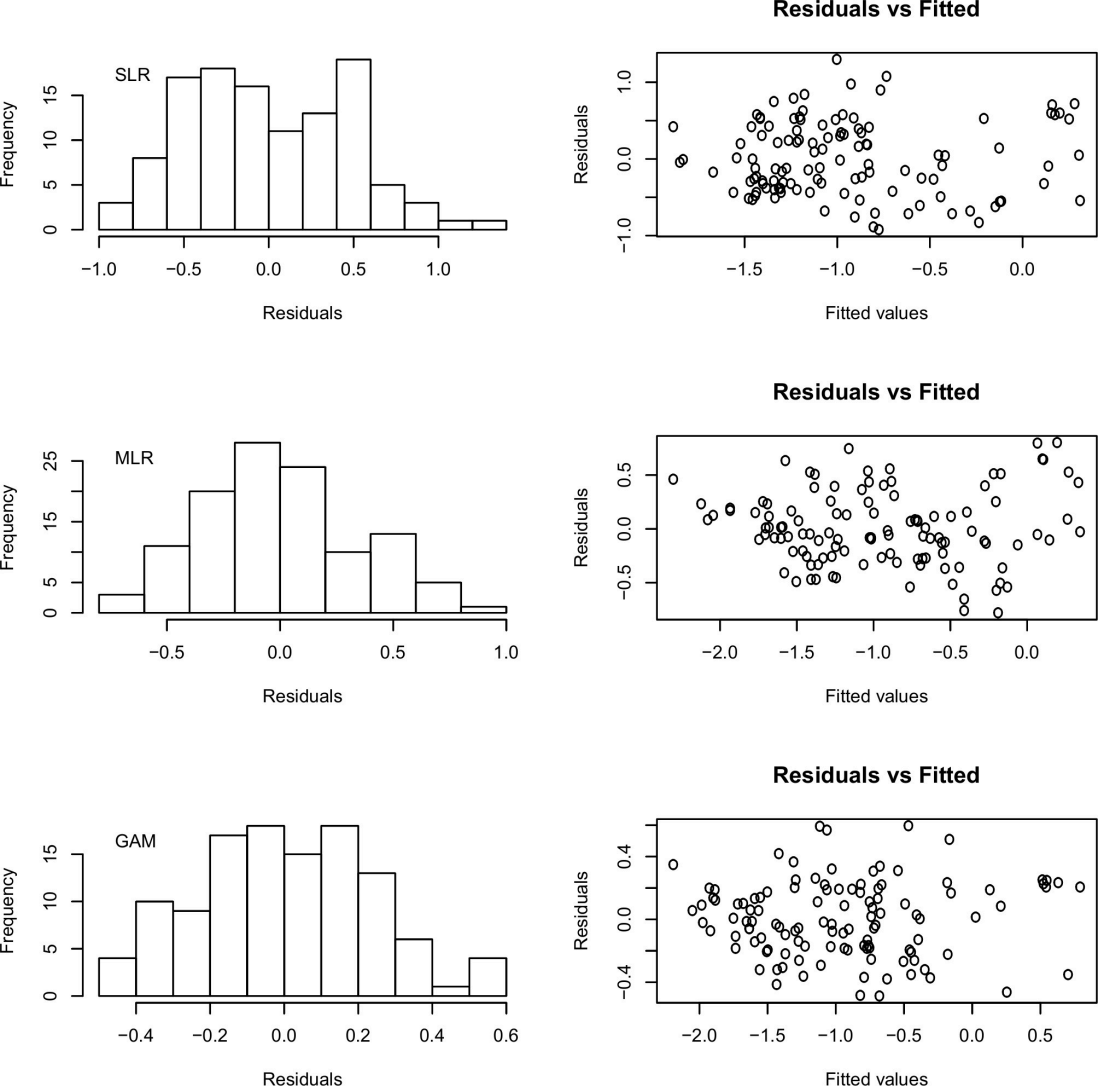
Residual analysis of the best fitted SLR (top), MLR (center), and GAM (bottom), respectively.

Fig 3 shows residual patterns of best fitted SLR, MLR, and GAM, respectively. As it can be observed, residuals of the three models seemed to have normal distribution with mean 0, no marked trend (homoscedasticity) in residuals versus fitted, and absence of outliers. Therefore, model assumptions seemed to be fine.

### Optimal model for estimating Chl-a

Given the results of Tables 2 and 3, the MLR with four bands (B1 [coastal/aerosol], B2 [blue], B3 [green], and B5 [NIR]) was considered as the best model to predict log-transformed Chl-a in the study area. Table 4 shows coefficients of this model, from which, we could infer that log-transformed Chl-a had a positive linear relationship with bands B1, B3, and B5 and negative linear relationship with band B2. Coefficients of this model suggested that B2, B3, and B1 had the strongest linear relationship with log-transformed Chl-a; on the contrary B5 had the weakest linear relationship with log-transformed Chl-a.

**Table 4:** Descriptive statistics of coefficients of the best-fitted model.

	Coefficient	Standard error	T value	P
Intercept	1.54	0.77	2.13	0.036
B1 (c/a)	79.56	14.96	5.32	<0.001
B2 (blue)	-191.62	15.55	-11.58	<0.001
B3 (green)	102.22	6.65	15.36	<0.001
B5 (NIR)	13.17	3.70	3.56	<0.001

### Temporal variability of predicted Chlorophyll-a

**Fig 4:**
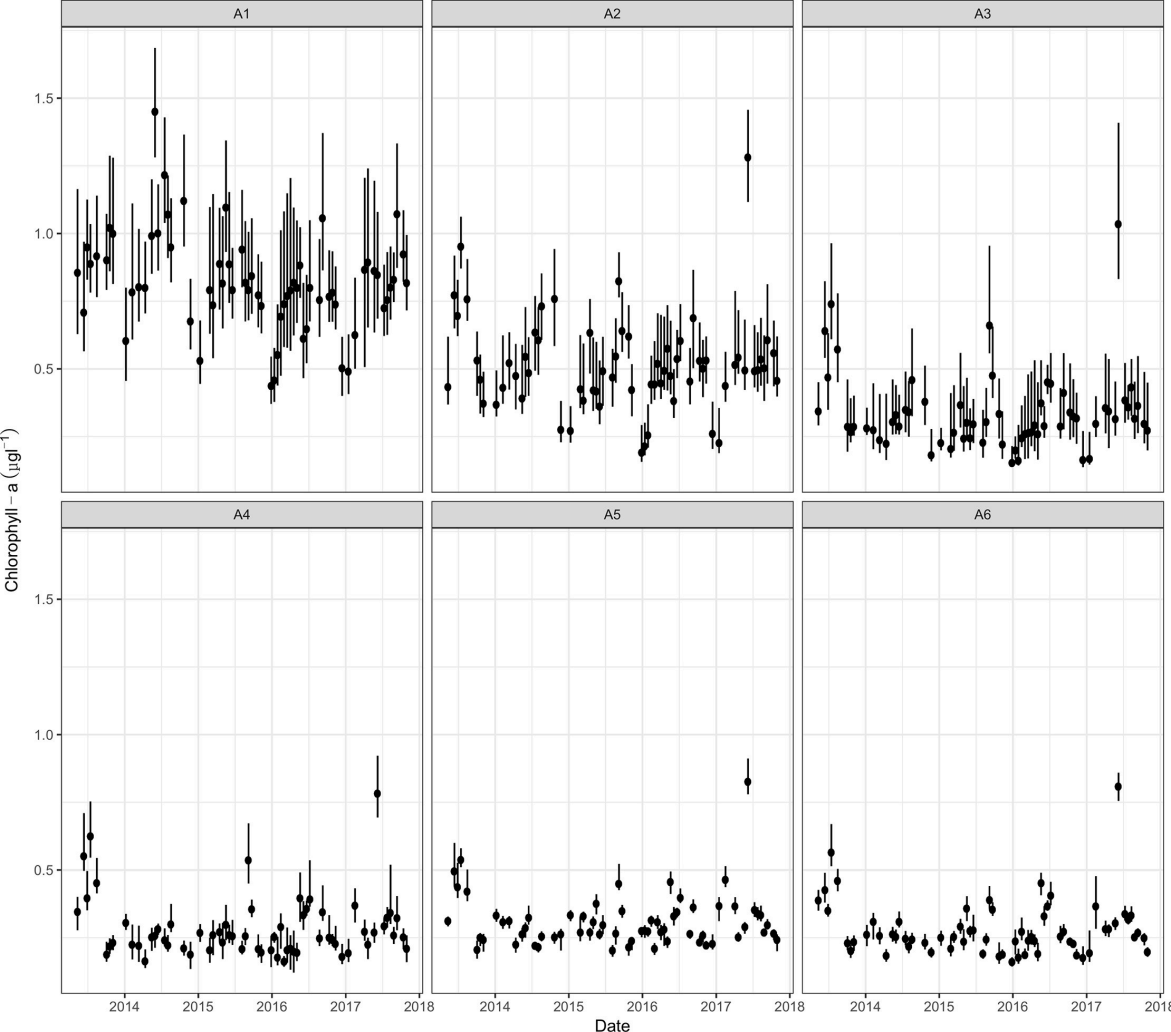
Predictions of Chl-a (μg*l^-1^) obtained from the best-fitted model and the Landsat imagery set for the period 2013–2017. Points represent the means; whiskers represent the interquartile ranges. A1-A6 arbitrary areas (see Fig 1 for details).

Predicted values of Chl-a for the period May 2013—October 2017 within the six arbitrary areas defined in this study are shown in Fig 4, ranging from 0.11 to 1.58 μg * l^−1^ and displaying high seasonal variability with peaks of maximum Chl-a during May and June; the lowest values were predicted for December and January and the highest ones corresponded to the red tide event observed in June 8, 2017; however, it is important to notice from model predictions, that this event was not present in area 1 (Ensenada de La Paz).

### Spatial variability of predicted Chlorophyll-a

**Fig 5:**
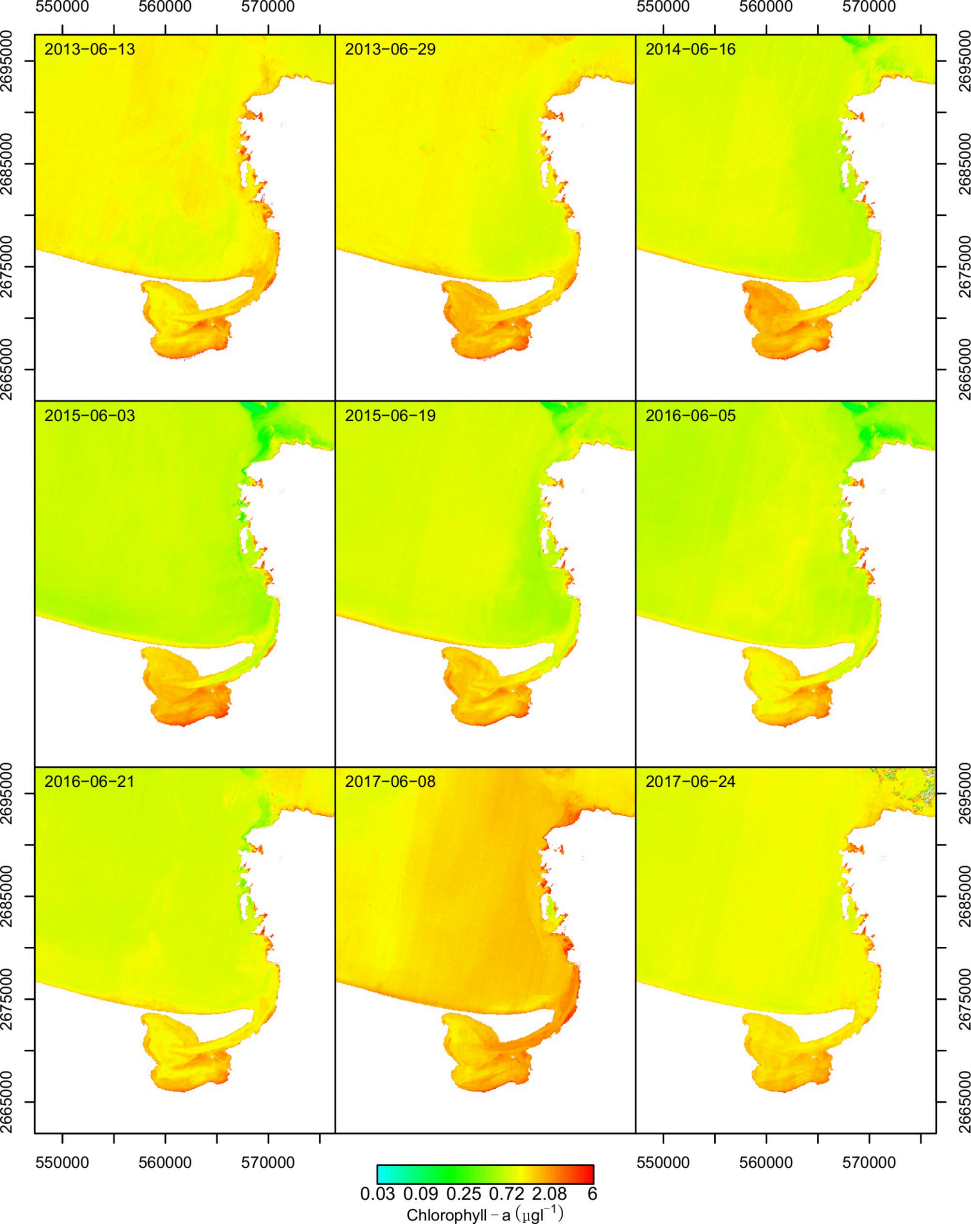
Predicted Chlorophyll-a (μg*l^-1^) in the study area, corresponding to June 2013 to 2017. Maps generated in programming language R.

Fig 5 represents the predicted Chl-a concentration of the study area corresponding to the month of June from 2013 to 2017 where the values obtained in each of the dates were compared; high values were observed in 2017-06-08 because this image showed conditions detected during a proliferation event registered in the study area. In the same way, high values were observed in the image corresponding to 2013-06-13 compared with the rest of the images because they corresponded to values prior to the proliferation event reported in literature from June 18 to 20, 2013. It indicated that based on the data obtained *in situ* during a year of monitoring, it was possible to set reference values through those generated by the model; Chl-a anomalies can be detected and can be used as indicators of possible algal proliferation events.

## Discussion

This study evaluated the use of Landsat 8 for estimating Chl-a concentration in the coastal water body located in northwestern Mexico by field data collection, simple linear regression, multiple linear regression and generalized additive models, using as response variable log-transformed Chl-a and reflectance values as spectral predictive variables of the visible part of light and NIR. The results obtained suggested that the use of spectral bands 1 (coastal/aerosol), 2 (blue), 3 (green), and 5 (NIR), from the MLR model, allowed us to reliably estimate the concentrations of Chl-a in a coastal environment.

To date, a large number of available publications have demonstrated that Chl-a can be estimated using Landsat satellite images by *in situ* data collection and using simple or multiple linear regression models, but something that attracted our attention was the great diversity of approaches that have been used for this purpose. For example, some authors have used simple models where the predictive variable was one of the spectral bands [31,32,46]; other authors suggested that the ratio of two spectral bands could be used as a good predictor of Chl-a [18,28,29,45,47]; finally, other authors suggested that various combinations of spectral bands in multiple linear regression models allowed a better estimation of Chl-a in aquatic environments [23,32,45–47].

These approaches generated uncertainty as to which was the best one to estimate Chl-a in aquatic environments. This study used a statistical approach and the Chl-a absorption/reflection theory to create the best possible model, in such a way, that MLR was constructed using spectral bands where Chl-a had its greater absorption/reflection. According to what several researchers have demonstrated, Chl-a had its highest light absorption at wavelengths from 400–500 nm (blue) and 680 nm (red) and its maximum reflection up to 550 nm (green) and 700 nm (NIR). Thus, a negative correlation was expected between Chl-a and reflectance in the blue band; that is, the higher the concentration of Chl-a, the lower reflectance in this wavelength. On the other hand, a positive correlation between Chl-a and reflectance in the green and NIR bands was expected; in other words, the higher the concentration of Chl-a, the higher reflectance in these wavelengths [45,48–51].

Initially, this study evaluated the linear correlation between log-transformed Chl-a and spectral reflectance in five wavelengths (coastal/aerosol, blue, green, red, and NIR) using Pearson correlation coefficients; however, two things that attracted our attention in the results obtained were (1) low correlation (r < 0.2) between Chl-a and the selected spectral bands and (2) correlation between Chl-a and the red and NIR bands, which had an opposite sign than that expected. In this regard, several authors have found higher or lower values of Pearson correlation coefficient and Chl-a; for example, Lim & Choi [44] found correlation values greater than 0.6 among the blue, green, red, and NIR bands and Chl-a; however, their results suggested inverse relationships because all correlation values were negative. On the other hand, Patra et al. [45] found correlations smaller than 0.5 and positive among blue, green, red and NIR bands and Chl-a. In both cases, the estimation of Chl-a was performed in freshwater bodies (rivers and lakes), which could have generated these differences with what was found in our study. Usually in freshwater bodies, such as rivers and lakes, turbidity (caused by particulate organic matter) is several times greater than in marine bodies [15,52,53].

Other authors have suggested that the combination of spectral bands by way of ratio (e.g. NIR/red) had a higher correlation with Chl-a [18,44–46,54]. In this regard, our study found higher correlation values between Chl-a and the red and squared transformed coastal/aerosol (B4/B12) band ratio. Another interesting point in this study was the use of the coastal aerosol band (B1) because when it was included, the models increased the correlation value (R) and decreased the value of RMSE. This band was constituted by wavelengths that detect deep blue and violet very similar to the blue band characterized by low reflectance in environments with high Chl-a concentration. According to Slonecker et al. [26] and Loyd [55], this feature makes the band potentially important for investigating coastal phenomena.

As mentioned above, the selected MLR was used to perform statistical inference, in this particular case, to evaluate the linear relationship between spectral bands and Chl-a by using the coefficients of multiple linear regression models. Our results showed a high concordance between the observed (model) and expected (absorption/reflection properties of Chl-a) results, specifically the negative coefficient of the blue (B2) band and positive coefficients of the green (B3) and NIR (B5) bands. In this regard, Brivio et al. [48] and Lim & Choi [47] used multiple linear regression models to estimate Chl-a (among others) through the use of different Landsat spectral bands; however, the coefficients of the best model used to estimate Chl-a showed the opposite expected signs. For example, positive values with the blue (B2) and negative with green (B3) bands.

To date, many studies have addressed estimating Chl-a from images acquired by satellites, both in freshwater bodies and marine environments, obtaining promising results. Nonetheless, when comparing methods and results, they have shown a great discrepancy in the way Chl-a has been estimated from Landsat images; it may be due to the wavelength used (or proportions among them), the type of statistical method, or the type of environment where the study was performed. All indicates that the methods applied in a specific place or environment cannot be replicated similarly in another place and/or different environment, which suggests the need for greater field validation and spatial or temporal coverage, or plainly and simply a comparison of Chl-a estimation with a standardized method in different types of the aquatic environments required.

## Conclusions

This study has evaluated the performance of simple and multiple linear regression and generalized additive models to estimate Chl-a concentration, using the first five bands of Landsat 8 images in the Bahía de La Paz, Baja California Sur, Mexico. The obtained results indicated that this method provided a reliable estimation of Chl-a in small coastal water bodies because of the high coherence found in model coefficients with the absorption/reflection properties of Chl-a evaluated in the laboratory under controlled conditions. Therefore, remote sensing has shown to represent an ideal opportunity to develop regional scale research on various parameters in environments estimated in small coastal water bodies to allow a constant monitoring at low cost and high-quality spatial scale.

## Supporting information

S1 Table Goodness of fit of the SLR models. (DOCX)

S2 Table Goodness of fit of the MLR models. (DOCX)

S3 Table Goodness of fit of the GAM models. (DOCX)

S4 Table Predictive performance of the SLR models. (DOCX)

S5 Table Predictive performance of the MLR models. (DOCX)

S6 Table Predictive performance of the GAM models. (DOCX)
