# Supplementary material for: Predictive performance of regression models to estimate Chlorophyll-a concentration based on Landsat imagery
Source: PLoS One. 2018 Oct 12;13(10):e0205682. doi: 10.1371/journal.pone.0205682 (PMC6185857; doi:10.1371/journal.pone.0205682)
Supplement: S3 Table — (DOCX) [file pone.0205682.s003.docx]

**S3 Table. Goodness of fit of the GAM models.**

| Model | R^2^ | Adjusted R^2^ |
| --- | --- | --- |
| y = f(B1) |  | 0.23 |
| y = f(B2) |  | 0.22 |
| y = f(B3) |  | 0.41 |
| y = f(B4) |  | 0.40 |
| y = f(B5) |  | 0.43 |
| y = f(B1) + f(B2) |  | 0.23 |
| y = f(B1) + f(B3) |  | 0.50 |
| y = f(B1) + f(B4) |  | 0.60 |
| y = f(B1) + f(B5) |  | 0.44 |
| y = f(B2) + f(B3) |  | 0.67 |
| y = f(B2) + f(B4) |  | 0.50 |
| y = f(B2) + f(B5) |  | 0.52 |
| y = f(B3) + f(B4) |  | 0.55 |
| y = f(B3) + f(B5) |  | 0.58 |
| y = f(B4) + f(B5) |  | 0.56 |
| y = f(B1) + f(B2) + f(B3) |  | 0.78 |
| y = f(B1) + f(B2) + f(B4) |  | 0.61 |
| y = f(B1) + f(B2) + f(B5) |  | 0.58 |
| y = f(B1) + f(B3) + f(B4) |  | 0.71 |
| y = f(B1) + f(B3) + f(B5) |  | 0.71 |
| y = f(B1) + f(B4) + f(B5) |  | 0.70 |
| y = f(B2) + f(B3) + f(B4) |  | 0.82 |
| y = f(B2) + f(B3) + f(B5) |  | 0.82 |
| y = f(B2) + f(B4) + f(B5) |  | 0.68 |
| y = f(B3) + f(B4) + f(B5) |  | 0.66 |
| y = f(B1) + f(B2) + f(B3) + f(B4) |  | 0.85 |
| y = f(B1) + f(B2) + f(B3) + f(B5) |  | 0.85 |
| y = f(B1) + f(B2) + f(B4) + f(B5) |  | 0.70 |
| y = f(B1) + f(B3) + f(B4) + f(B5) |  | 0.75 |
| y = f(B2) + f(B3) + f(B4) + f(B5) |  | 0.83 |
| y = f(B1) + f(B2) + f(B3) + f(B4) + f(B5) |  | 0.85 |
