# Supplementary material for: Predictive performance of regression models to estimate Chlorophyll-a concentration based on Landsat imagery
Source: PLoS One. 2018 Oct 12;13(10):e0205682. doi: 10.1371/journal.pone.0205682 (PMC6185857; doi:10.1371/journal.pone.0205682)
Supplement: S4 Table — (DOCX) [file pone.0205682.s004.docx]

**S4 Table. Predictive performance of the SLR models.**

| Model | R | MSRE |
| --- | --- | --- |
| y = -2.48 + 11.72*(B1) | 0.39 | 0.67 |
| y = -1.76 + 7.35*(B2) | 0.25 | 0.67 |
| y = -1.71 + 9.89*(B3) | 0.39 | 0.61 |
| y = -2.08 + 26.69*(B4) | 0.78 | 0.48 |
| y = -1.54 + 25.69*(B5) | 0.75 | 0.64 |
| y = -2.48 + 11.72*(B1^2^) | 0.39 | 0.67 |
| y = -1.76 + 7.35*(B2^2^) | 0.25 | 0.67 |
| y = -1.71 + 9.89*(B3^2^) | 0.39 | 0.61 |
| y = -2.08 + 26.69*(B4^2^) | 0.78 | 0.48 |
| y = -1.54 + 25.69*(B5^2^) | 0.75 | 0.64 |
| y = -2.06 + 4.62*(B1+B2) | 0.31 | 0.67 |
| y = -2.1 + 8.03*(B1+B2^2^) | 0.34 | 0.67 |
| y = -2.16 + 5.82*(B1+B3) | 0.39 | 0.63 |
| y = -2.19 + 9.04*(B1+B3^2^) | 0.36 | 0.66 |
| y = -2.84 + 10.95*(B1+B4) | 0.64 | 0.59 |
| y = -2.59 + 12.37*(B1+B4^2^) | 0.44 | 0.66 |
| y = -2.81 + 12.14*(B1+B5) | 0.66 | 0.65 |
| y = -2.58 + 12.44*(B1+B5^2^) | 0.44 | 0.67 |
| y = -1.73 + 6.17*(B1^2^+B2) | 0.27 | 0.67 |
| y = -1.37 + 14.18*(B1^2^+B2^2^) | 0.25 | 0.67 |
| y = -1.72 + 8.22*(B1^2^+B3) | 0.38 | 0.62 |
| y = -1.38 + 18.33*(B1^2^+B3^2^) | 0.29 | 0.66 |
| y = -2.12 + 19.58*(B1^2^+B4) | 0.72 | 0.53 |
| y = -1.74 + 41.23*(B1^2^+B4^2^) | 0.50 | 0.64 |
| y = -1.79 + 20.95*(B1^2^+B5) | 0.73 | 0.64 |
| y = -1.73 + 43.79*(B1^2^+B5^2^) | 0.48 | 0.67 |
| y = -2.06 + 4.62*(B2+B1) | 0.31 | 0.67 |
| y = -1.73 + 6.17*(B2+B1^2^) | 0.27 | 0.67 |
| y = -1.83 + 4.69*(B2+B3) | 0.33 | 0.64 |
| y = -1.67 + 6.18*(B2+B3^2^) | 0.25 | 0.66 |
| y = -2.27 + 8.67*(B2+B4) | 0.55 | 0.60 |
| y = -1.84 + 8.01*(B2+B4^2^) | 0.30 | 0.66 |
| y = -2.27 + 9.89*(B2+B5) | 0.58 | 0.63 |
| y = -1.83 + 7.94*(B2+B5^2^) | 0.29 | 0.66 |
| y = -2.1 + 8.03*(B2^2^+B1) | 0.34 | 0.67 |
| y = -1.37 + 14.18*(B2^2^+B1^2^) | 0.25 | 0.67 |
| y = -1.63 + 7.63*(B2^2^+B3) | 0.36 | 0.63 |
| y = -1.22 + 14.26*(B2^2^+B3^2^) | 0.21 | 0.67 |
| y = -1.96 + 18.48*(B2^2^+B4) | 0.70 | 0.54 |
| y = -1.38 + 29.9*(B2^2^+B4^2^) | 0.35 | 0.65 |
| y = -1.68 + 20.72*(B2^2^+B5) | 0.71 | 0.62 |
| y = -1.34 + 30.24*(B2^2^+B5^2^) | 0.32 | 0.66 |
| y = -2.16 + 5.82*(B3+B1) | 0.39 | 0.63 |
| y = -1.72 + 8.22*(B3+B1^2^) | 0.38 | 0.62 |
| y = -1.83 + 4.69*(B3+B2) | 0.33 | 0.64 |
| y = -1.63 + 7.63*(B3+B2^2^) | 0.36 | 0.63 |
| y = -1.95 + 8.37*(B3+B4) | 0.58 | 0.56 |
| y = -1.72 + 9.79*(B3+B4^2^) | 0.42 | 0.61 |
| y = -1.96 + 10.02*(B3+B5) | 0.62 | 0.55 |
| y = -1.73 + 10.08*(B3+B5^2^) | 0.41 | 0.61 |
| y = -2.19 + 9.04*(B3^2^+B1) | 0.36 | 0.66 |
| y = -1.38 + 18.33*(B3^2^+B1^2^) | 0.29 | 0.66 |
| y = -1.67 + 6.18*(B3^2^+B2) | 0.25 | 0.66 |
| y = -1.22 + 14.26*(B3^2^+B2^2^) | 0.21 | 0.67 |
| y = -1.87 + 18.79*(B3^2^+B4) | 0.71 | 0.52 |
| y = -1.25 + 35.68*(B3^2^+B4^2^) | 0.41 | 0.62 |
| y = -1.61 + 22.31*(B3^2^+B5) | 0.73 | 0.57 |
| y = -1.24 + 39.71*(B3^2^+B5^2^) | 0.38 | 0.63 |
| y = -2.84 + 10.95*(B4+B1) | 0.64 | 0.59 |
| y = -2.12 + 19.58*(B4+B1^2^) | 0.72 | 0.53 |
| y = -2.27 + 8.67*(B4+B2) | 0.55 | 0.60 |
| y = -1.96 + 18.48*(B4+B2^2^) | 0.70 | 0.54 |
| y = -1.95 + 8.37*(B4+B3) | 0.58 | 0.56 |
| y = -1.87 + 18.79*(B4+B3^2^) | 0.71 | 0.52 |
| y = -1.92 + 14.77*(B4+B5) | 0.78 | 0.50 |
| y = -2.05 + 25.52*(B4+B5^2^) | 0.78 | 0.48 |
| y = -2.59 + 12.37*(B4^2^+B1) | 0.44 | 0.66 |
| y = -1.74 + 41.23*(B4^2^+B1^2^) | 0.50 | 0.64 |
| y = -1.84 + 8.01*(B4^2^+B2) | 0.30 | 0.66 |
| y = -1.38 + 29.9*(B4^2^+B2^2^) | 0.35 | 0.65 |
| y = -1.72 + 9.79*(B4^2^+B3) | 0.42 | 0.61 |
| y = -1.25 + 35.68*(B4^2^+B3^2^) | 0.41 | 0.62 |
| y = -1.56 + 24.36*(B4^2^+B5) | 0.76 | 0.61 |
| y = -1.38 + 158.4*(B4^2^+B5^2^) | 0.79 | 0.54 |
| y = -2.81 + 12.14*(B5+B1) | 0.66 | 0.65 |
| y = -1.79 + 20.95*(B5+B1^2^) | 0.73 | 0.64 |
| y = -2.27 + 9.89*(B5+B2) | 0.58 | 0.63 |
| y = -1.68 + 20.72*(B5+B2^2^) | 0.71 | 0.62 |
| y = -1.96 + 10.02*(B5+B3) | 0.62 | 0.55 |
| y = -1.61 + 22.31*(B5+B3^2^) | 0.73 | 0.57 |
| y = -1.92 + 14.77*(B5+B4) | 0.78 | 0.50 |
| y = -1.56 + 24.36*(B5+B4^2^) | 0.76 | 0.61 |
| y = -2.58 + 12.44*(B5^2^+B1) | 0.44 | 0.67 |
| y = -1.73 + 43.79*(B5^2^+B1^2^) | 0.48 | 0.67 |
| y = -1.83 + 7.94*(B5^2^+B2) | 0.29 | 0.66 |
| y = -1.34 + 30.24*(B5^2^+B2^2^) | 0.32 | 0.66 |
| y = -1.73 + 10.08*(B5^2^+B3) | 0.41 | 0.61 |
| y = -1.24 + 39.71*(B5^2^+B3^2^) | 0.38 | 0.63 |
| y = -2.05 + 25.52*(B5^2^+B4) | 0.78 | 0.48 |
| y = -1.38 + 158.4*(B5^2^+B4^2^) | 0.79 | 0.54 |
| y = -1.36 + 28.13*(B1*B2) | 0.25 | 0.67 |
| y = -1.1 + 94.29*(B1*B2^2^) | 0.15 | 0.68 |
| y = -1.36 + 39.62*(B1*B3) | 0.32 | 0.64 |
| y = -1.08 + 143.37*(B1*B3^2^) | 0.19 | 0.67 |
| y = -1.65 + 122.39*(B1*B4) | 0.70 | 0.56 |
| y = -1.29 + 1219.67*(B1*B4^2^) | 0.71 | 0.58 |
| y = -1.43 + 155.11*(B1*B5) | 0.74 | 0.64 |
| y = -1.13 + 1888.99*(B1*B5^2^) | 0.78 | 0.66 |
| y = -1.18 + 117.45*(B1^2^*B2) | 0.21 | 0.68 |
| y = -1.04 + 402.94*(B1^2^*B2^2^) | 0.12 | 0.69 |
| y = -1.18 + 164.03*(B1^2^*B3) | 0.25 | 0.66 |
| y = -1.02 + 595.98*(B1^2^*B3^2^) | 0.13 | 0.68 |
| y = -1.38 + 556.16*(B1^2^*B4) | 0.60 | 0.61 |
| y = -1.18 + 5962.52*(B1^2^*B4^2^) | 0.63 | 0.61 |
| y = -1.33 + 902.94*(B1^2^*B5) | 0.72 | 0.65 |
| y = -1.09 + 10789.19*(B1^2^*B5^2^) | 0.77 | 0.67 |
| y = -1.36 + 28.13*(B2*B1) | 0.25 | 0.67 |
| y = -1.18 + 117.45*(B2*B1^2^) | 0.21 | 0.68 |
| y = -1.23 + 31.42*(B2*B3) | 0.24 | 0.66 |
| y = -1.04 + 115.23*(B2*B3^2^) | 0.13 | 0.68 |
| y = -1.49 + 112.13*(B2*B4) | 0.62 | 0.59 |
| y = -1.23 + 1170.52*(B2*B4^2^) | 0.66 | 0.60 |
| y = -1.41 + 174.83*(B2*B5) | 0.72 | 0.63 |
| y = -1.13 + 2178.52*(B2*B5^2^) | 0.77 | 0.66 |
| y = -1.1 + 94.29*(B2^2^*B1) | 0.15 | 0.68 |
| y = -1.04 + 402.94*(B2^2^*B1^2^) | 0.12 | 0.69 |
| y = -1.06 + 105.17*(B2^2^*B3) | 0.13 | 0.68 |
| y = -0.98 + 364.15*(B2^2^*B3^2^) | 0.04 | 0.69 |
| y = -1.2 + 442.79*(B2^2^*B4) | 0.43 | 0.64 |
| y = -1.1 + 5067.18*(B2^2^*B4^2^) | 0.49 | 0.64 |
| y = -1.28 + 1093.66*(B2^2^*B5) | 0.66 | 0.63 |
| y = -1.09 + 14182.23*(B2^2^*B5^2^) | 0.75 | 0.66 |
| y = -1.36 + 39.62*(B3*B1) | 0.32 | 0.64 |
| y = -1.18 + 164.03*(B3*B1^2^) | 0.25 | 0.66 |
| y = -1.23 + 31.42*(B3*B2) | 0.24 | 0.66 |
| y = -1.06 + 105.17*(B3*B2^2^) | 0.13 | 0.68 |
| y = -1.35 + 112.75*(B3*B4) | 0.59 | 0.58 |
| y = -1.17 + 1182.74*(B3*B4^2^) | 0.62 | 0.61 |
| y = -1.39 + 230.35*(B3*B5) | 0.73 | 0.56 |
| y = -1.13 + 2921.91*(B3*B5^2^) | 0.78 | 0.64 |
| y = -1.08 + 143.37*(B3^2^*B1) | 0.19 | 0.67 |
| y = -1.02 + 595.98*(B3^2^*B1^2^) | 0.13 | 0.68 |
| y = -1.04 + 115.23*(B3^2^*B2) | 0.13 | 0.68 |
| y = -0.98 + 364.15*(B3^2^*B2^2^) | 0.04 | 0.69 |
| y = -1.09 + 431.08*(B3^2^*B4) | 0.36 | 0.65 |
| y = -1.03 + 4629.09*(B3^2^*B4^2^) | 0.39 | 0.66 |
| y = -1.24 + 1685.02*(B3^2^*B5) | 0.61 | 0.58 |
| y = -1.09 + 24842.88*(B3^2^*B5^2^) | 0.74 | 0.64 |
| y = -1.65 + 122.39*(B4*B1) | 0.70 | 0.56 |
| y = -1.38 + 556.16*(B4*B1^2^) | 0.60 | 0.61 |
| y = -1.49 + 112.13*(B4*B2) | 0.62 | 0.59 |
| y = -1.2 + 442.79*(B4*B2^2^) | 0.43 | 0.64 |
| y = -1.35 + 112.75*(B4*B3) | 0.59 | 0.58 |
| y = -1.09 + 431.08*(B4*B3^2^) | 0.36 | 0.65 |
| y = -1.32 + 326.22*(B4*B5) | 0.79 | 0.57 |
| y = -1.1 + 3879.19*(B4*B5^2^) | 0.80 | 0.64 |
| y = -1.29 + 1219.67*(B4^2^*B1) | 0.71 | 0.58 |
| y = -1.18 + 5962.52*(B4^2^*B1^2^) | 0.63 | 0.61 |
| y = -1.23 + 1170.52*(B4^2^*B2) | 0.66 | 0.60 |
| y = -1.1 + 5067.18*(B4^2^*B2^2^) | 0.49 | 0.64 |
| y = -1.17 + 1182.74*(B4^2^*B3) | 0.62 | 0.61 |
| y = -1.03 + 4629.09*(B4^2^*B3^2^) | 0.39 | 0.66 |
| y = -1.19 + 3950.63*(B4^2^*B5) | 0.80 | 0.58 |
| y = -1.05 + 43747.58*(B4^2^*B5^2^) | 0.80 | 0.65 |
| y = -1.43 + 155.11*(B5*B1) | 0.74 | 0.64 |
| y = -1.33 + 902.94*(B5*B1^2^) | 0.72 | 0.65 |
| y = -1.41 + 174.83*(B5*B2) | 0.72 | 0.63 |
| y = -1.28 + 1093.66*(B5*B2^2^) | 0.66 | 0.63 |
| y = -1.39 + 230.35*(B5*B3) | 0.73 | 0.56 |
| y = -1.24 + 1685.02*(B5*B3^2^) | 0.61 | 0.58 |
| y = -1.32 + 326.22*(B5*B4) | 0.79 | 0.57 |
| y = -1.19 + 3950.63*(B5*B4^2^) | 0.80 | 0.58 |
| y = -1.13 + 1888.99*(B5^2^*B1) | 0.78 | 0.66 |
| y = -1.09 + 10789.19*(B5^2^*B1^2^) | 0.77 | 0.67 |
| y = -1.13 + 2178.52*(B5^2^*B2) | 0.77 | 0.66 |
| y = -1.09 + 14182.23*(B5^2^*B2^2^) | 0.75 | 0.66 |
| y = -1.13 + 2921.91*(B5^2^*B3) | 0.78 | 0.64 |
| y = -1.09 + 24842.88*(B5^2^*B3^2^) | 0.74 | 0.64 |
| y = -1.1 + 3879.19*(B5^2^*B4) | 0.80 | 0.64 |
| y = -1.05 + 43747.58*(B5^2^*B4^2^) | 0.80 | 0.65 |
| y = 1.4 - 1.96*(B1/B2) | 0.12 | 0.69 |
| y = 0.11 - 0.09*(B1/B2^2^) | 0.35 | 0.65 |
| y = 1.06 - 1.13*(B1/B3) | 0.55 | 0.52 |
| y = -0.03 - 0.04*(B1/B3^2^) | 0.59 | 0.49 |
| y = 1.08 - 0.61*(B1/B4) | 0.78 | 0.35 |
| y = -0.07 - 0.01*(B1/B4^2^) | 0.72 | 0.39 |
| y = -0.1 - 0.12*(B1/B5) | 0.65 | 0.67 |
| y = -0.53 - 0*(B1/B5^2^) | 0.59 | 0.69 |
| y = -3.39 + 15.69*(B1^2^/B2) | -0.59 | 0.78 |
| y = 0.37 - 0.92*(B1^2^/B2^2^) | 0.14 | 0.69 |
| y = 2 - 12.85*(B1^2^/B3) | 0.50 | 0.59 |
| y = 0.14 - 0.33*(B1^2^/B3^2^) | 0.58 | 0.50 |
| y = 1.84 - 6.54*(B1^2^/B4) | 0.79 | 0.29 |
| y = 0.09 - 0.09*(B1^2^/B4^2^) | 0.74 | 0.35 |
| y = -0.22 - 0.82*(B1^2^/B5) | 0.63 | 0.69 |
| y = -0.53 - 0.01*(B1^2^/B5^2^) | -0.59 | 0.69 |
| y = -2.72 + 2.12*(B2/B1) | 0.08 | 0.70 |
| y = 1.83 - 0.43*(B2/B1^2^) | -0.61 | 0.80 |
| y = 2.11 - 2.07*(B2/B3) | 0.65 | 0.44 |
| y = 0.1 - 0.05*(B2/B3^2^) | 0.61 | 0.47 |
| y = 1.27 - 0.8*(B2/B4) | 0.77 | 0.31 |
| y = -0.03 - 0.01*(B2/B4^2^) | 0.73 | 0.38 |
| y = -0.19 - 0.13*(B2/B5) | -0.63 | 0.72 |
| y = -0.55 - 0*(B2/B5^2^) | -0.59 | 0.72 |
| y = -1.42 + 5.04*(B2^2^/B1) | 0.17 | 0.68 |
| y = -1.7 + 1.07*(B2^2^/B1^2^) | 0.06 | 0.70 |
| y = 4.33 - 32.94*(B2^2^/B3) | 0.63 | 0.47 |
| y = 0.64 - 0.71*(B2^2^/B3^2^) | 0.65 | 0.44 |
| y = 0.84 - 5.95*(B2^2^/B4) | 0.61 | 0.44 |
| y = 0.11 - 0.13*(B2^2^/B4^2^) | 0.71 | 0.34 |
| y = -0.56 - 0.61*(B2^2^/B5) | -0.55 | 0.72 |
| y = -0.63 - 0.01*(B2^2^/B5^2^) | -0.57 | 0.72 |
| y = -2.47 + 2.61*(B3/B1) | 0.45 | 0.57 |
| y = -3.77 + 0.64*(B3/B1^2^) | 0.45 | 0.62 |
| y = -3.72 + 4.02*(B3/B2) | 0.61 | 0.48 |
| y = -6.38 + 0.86*(B3/B2^2^) | 0.64 | 0.46 |
| y = 0.41 - 0.72*(B3/B4) | -0.52 | 0.72 |
| y = -0.01 - 0.02*(B3/B4^2^) | 0.68 | 0.46 |
| y = -0.67 - 0.07*(B3/B5) | -0.47 | 0.81 |
| y = -0.67 - 0*(B3/B5^2^) | -0.52 | 0.82 |
| y = -1.31 + 7.68*(B3^2^/B1) | 0.31 | 0.64 |
| y = -1.55 + 1.71*(B3^2^/B1^2^) | 0.37 | 0.61 |
| y = -1.47 + 9.46*(B3^2^/B2) | 0.40 | 0.61 |
| y = -2.24 + 2.66*(B3^2^/B2^2^) | 0.58 | 0.50 |
| y = -1.02 + 0.54*(B3^2^/B4) | -0.07 | 0.75 |
| y = -0.42 - 0.15*(B3^2^/B4^2^) | -0.44 | 0.69 |
| y = -0.96 + 0.03*(B3^2^/B5) | -0.20 | 0.72 |
| y = -0.89 - 0*(B3^2^/B5^2^) | -0.38 | 0.73 |
| y = -2.77 + 5.68*(B4/B1) | 0.84 | 0.36 |
| y = -3.6 + 1.09*(B4/B1^2^) | 0.86 | 0.26 |
| y = -3.06 + 5.55*(B4/B2) | 0.85 | 0.30 |
| y = -3.45 + 0.72*(B4/B2^2^) | 0.77 | 0.31 |
| y = -2.72 + 3.23*(B4/B3) | -0.65 | 0.75 |
| y = -0.26 - 0.09*(B4/B3^2^) | 0.13 | 0.71 |
| y = -0.84 - 0.05*(B4/B5) | -0.41 | 0.77 |
| y = -0.65 - 0*(B4/B5^2^) | -0.54 | 0.80 |
| y = -1.6 + 44.39*(B4^2^/B1) | 0.82 | 0.45 |
| y = -1.8 + 7.84*(B4^2^/B1^2^) | 0.85 | 0.37 |
| y = -1.68 + 42.26*(B4^2^/B2) | 0.84 | 0.42 |
| y = -1.94 + 6.54*(B4^2^/B2^2^) | 0.87 | 0.31 |
| y = -1.81 + 35.85*(B4^2^/B3) | 0.82 | 0.47 |
| y = -1.88 + 3.01*(B4^2^/B3^2^) | -0.69 | 0.75 |
| y = -1.16 + 2.49*(B4^2^/B5) | 0.35 | 0.67 |
| y = -0.95 - 0*(B4^2^/B5^2^) | -0.33 | 0.72 |
| y = -1.66 + 4.09*(B5/B1) | 0.75 | 0.63 |
| y = -1.77 + 0.62*(B5/B1^2^) | 0.73 | 0.64 |
| y = -1.67 + 3.48*(B5/B2) | 0.74 | 0.68 |
| y = -1.74 + 0.42*(B5/B2^2^) | -0.71 | 0.78 |
| y = -1.5 + 1.84*(B5/B3) | -0.64 | 0.98 |
| y = -0.86 - 0.02*(B5/B3^2^) | -0.31 | 1.02 |
| y = -1.48 + 1.01*(B5/B4) | -0.54 | 0.97 |
| y = 0.53 - 0.11*(B5/B4^2^) | 0.57 | 0.51 |
| y = -1.21 + 52.27*(B5^2^/B1) | 0.78 | 0.65 |
| y = -1.26 + 8.27*(B5^2^/B1^2^) | 0.78 | 0.64 |
| y = -1.22 + 44.87*(B5^2^/B2) | 0.78 | 0.66 |
| y = -1.26 + 6.04*(B5^2^/B2^2^) | 0.78 | 0.67 |
| y = -1.21 + 31.85*(B5^2^/B3) | -0.76 | 0.72 |
| y = -1.22 + 2.67*(B5^2^/B3^2^) | -0.70 | 0.90 |
| y = -1.27 + 23.38*(B5^2^/B4) | -0.73 | 0.73 |
| y = -1.27 + 1.09*(B5^2^/B4^2^) | -0.59 | 0.97 |
