# Supplementary material for: Predictive performance of regression models to estimate Chlorophyll-a concentration based on Landsat imagery
Source: PLoS One. 2018 Oct 12;13(10):e0205682. doi: 10.1371/journal.pone.0205682 (PMC6185857; doi:10.1371/journal.pone.0205682)
Supplement: S5 Table — (DOCX) [file pone.0205682.s005.docx]

**S2 Table. Predictive performance of the MLR models.**

| Model | R | MSRE |
| --- | --- | --- |
| y = -3.64 + 36.92*B1 - 19.37*B2 | -0.42 | 0.97 |
| y = 1.42 - 33.97*B1 + 26.77*B3 | 0.16 | 0.77 |
| y = 2.34 - 44.69*B1 + 60.04*B4 | 0.79 | 0.33 |
| y = -1.24 - 2.59*B1 + 27.09*B5 | 0.70 | 0.64 |
| y = 4.25 - 107.06*B2 + 85.15*B3 | 0.72 | 0.42 |
| y = -0.29 - 25.43*B2 + 50.34*B4 | 0.79 | 0.31 |
| y = -1.48 - 0.63*B2 + 26.05*B5 | 0.29 | 0.66 |
| y = -1.89 - 9.58*B3 + 39.54*B4 | 0.67 | 0.53 |
| y = -1.85 + 5.19*B3 + 21.76*B5 | 0.18 | 0.67 |
| y = -2.05 + 23.16*B4 + 5.1*B5 | 0.56 | 0.58 |
| y = 0.18 + 114.07*B1 - 222.66*B2 + 109.74*B3 | 0.86 | 0.21 |
| y = 3.11 - 60.3*B1 + 10.92*B2 + 61.54*B4 | 0.85 | 0.47 |
| y = 0.22 - 32.34*B1 + 21.19*B2 + 31.22*B5 | 0.11 | 0.88 |
| y = 4.55 - 70.67*B1 + 17.44*B3 + 56.01*B4 | 0.85 | 0.45 |
| y = 4.65 - 71.61*B1 + 37.75*B3 + 35.75*B5 | 0.72 | 0.58 |
| y = 2.69 - 48.94*B1 + 70.49*B4 - 10.54*B5 | 0.77 | 0.37 |
| y = 3.74 - 100.72*B2 + 63.31*B3 + 35.33*B4 | 0.86 | 0.29 |
| y = 4.51 - 114.8*B2 + 84.99*B3 + 25.93*B5 | 0.88 | 0.28 |
| y = 0.03 - 31.35*B2 + 67.09*B4 - 16.28*B5 | 0.71 | 0.37 |
| y = -1.88 - 13.13*B3 + 51.03*B4 - 9.72*B5 | 0.72 | 0.54 |
| y = 0.94 + 88.45*B1 - 194.77*B2 + 97.55*B3 + 10.79*B4 | 0.84 | 0.22 |
| y = 1.54 + 79.56*B1 - 191.62*B2 + 102.22*B3 + 13.17*B5 | 0.88 | 0.19 |
| y = 2.83 - 51.95*B1 + 2.27*B2 + 70.22*B4 - 9.95*B5 | 0.83 | 0.44 |
| y = 4.67 - 71.99*B1 + 19.4*B3 + 51.59*B4 + 4*B5 | 0.85 | 0.47 |
| y = 4.16 - 108.52*B2 + 74.49*B3 + 17.09*B4 + 15.16*B5 | 0.88 | 0.28 |
| y = 1.05 + 103.37*B1 - 221.63*B2 + 119.1*B3 - 19.09*B4 + 21.39*B5 | 0.87 | 0.19 |
