# Supplementary material for: Predictive performance of regression models to estimate Chlorophyll-a concentration based on Landsat imagery
Source: PLoS One. 2018 Oct 12;13(10):e0205682. doi: 10.1371/journal.pone.0205682 (PMC6185857; doi:10.1371/journal.pone.0205682)
Supplement: S6 Table — (DOCX) [file pone.0205682.s006.docx]

**S3 Table. Predictive performance of the GAM models.**

| Model | R | MSRE |
| --- | --- | --- |
| y = f(B1) | -0.15 | 0.87 |
| y = f(B2) | 0.26 | 0.65 |
| y = f(B3) | 0.69 | 0.43 |
| y = f(B4) | 0.76 | 0.45 |
| y = f(B5) | 0.75 | 0.64 |
| y = f(B1) + f(B2) | -0.28 | 1.26 |
| y = f(B1) + f(B3) | 0.56 | 0.66 |
| y = f(B1) + f(B4) | 0.66 | 0.56 |
| y = f(B1) + f(B5) | -0.15 | 0.88 |
| y = f(B2) + f(B3) | 0.77 | 0.34 |
| y = f(B2) + f(B4) | 0.70 | 0.36 |
| y = f(B2) + f(B5) | -0.16 | 0.83 |
| y = f(B3) + f(B4) | 0.60 | 0.59 |
| y = f(B3) + f(B5) | 0.47 | 0.65 |
| y = f(B4) + f(B5) | 0.43 | 0.60 |
| y = f(B1) + f(B2) + f(B3) | 0.84 | 0.24 |
| y = f(B1) + f(B2) + f(B4) | 0.65 | 0.70 |
| y = f(B1) + f(B2) + f(B5) | -0.34 | 1.37 |
| y = f(B1) + f(B3) + f(B4) | 0.76 | 0.53 |
| y = f(B1) + f(B3) + f(B5) | 0.54 | 0.75 |
| y = f(B1) + f(B4) + f(B5) | 0.43 | 0.59 |
| y = f(B2) + f(B3) + f(B4) | 0.82 | 0.29 |
| y = f(B2) + f(B3) + f(B5) | 0.76 | 0.38 |
| y = f(B2) + f(B4) + f(B5) | 0.36 | 0.65 |
| y = f(B3) + f(B4) + f(B5) | 0.31 | 0.69 |
| y = f(B1) + f(B2) + f(B3) + f(B4) | 0.80 | 0.27 |
| y = f(B1) + f(B2) + f(B3) + f(B5) | 0.75 | 0.32 |
| y = f(B1) + f(B2) + f(B4) + f(B5) | 0.36 | 0.71 |
| y = f(B1) + f(B3) + f(B4) + f(B5) | 0.56 | 0.61 |
| y = f(B2) + f(B3) + f(B4) + f(B5) | 0.75 | 0.37 |
| y = f(B1) + f(B2) + f(B3) + f(B4) + f(B5) | 0.75 | 0.32 |
